# Low-Fat and High-Quality Fermented Sausages

**DOI:** 10.3390/microorganisms8071025

**Published:** 2020-07-10

**Authors:** Patrizio Tremonte, Gianfranco Pannella, Silvia Jane Lombardi, Massimo Iorizzo, Franca Vergalito, Autilia Cozzolino, Lucia Maiuro, Mariantonietta Succi, Elena Sorrentino, Raffaele Coppola

**Affiliations:** Department of Agricultural, Environmental and Food Sciences (DiAAA), University of Molise, 86100 Campobasso, Italy; tremonte@unimol.it (P.T.); gianfranco.pannella@unimol.it (G.P.); silvia.lombardi@unimol.it (S.J.L.); franca.vergalito@unimol.it (F.V.); autilia.cozzolino@unimol.it (A.C.); maiuro@unimol.it (L.M.); succi@unimol.it (M.S.); sorrentino@unimol.it (E.S.); coppola@unimol.it (R.C.)

**Keywords:** lemon albedo, *Lactiplantibacillus plantarum*, antimicrobial activity, bio-preservation, fat replacement, anti-*Listeria* activity

## Abstract

The present study, considering for the first time microbiological concerns due to the use of lemon albedo as a fat replacer, aimed at the selection of an anti-*Listeria* strain to be used as protective culture in low-fat southern Italian fermented sausages. In fact, these kinds of products require appropriate bio-protective strategies to avoid risks due to *Listeria monocytogenes*. Sixty-seven *Lactiplantibacillus plantarum* strains isolated from diverse sources were screened for their antimicrobial activity and their interaction with starter strains (*Latilactobacillus sakei* 152 and *Staphylococcus xylosus* MVS9). *Lactiplantibacillus plantarum* Lpls100, highlighting both listericidal activity and the ability to promote *Staphylococcus xylosus* MVS9 growth, was used as a protective strain in low-fat fermented sausages prepared with lemon albedo as a fat replacer. The effect of the albedo and the protective strain on the fermentation process and the final quality was ascertained. Results highlighted that the use of the albedo did not affect the growth of starter strains and enhanced some quality features, such as fatty acid profiles and certain sensory attributes. However, the albedo also produced a slow decrease in water activity, compromising the microbial quality. The anti-*Listeria* strain, enhancing coagulase negative cocci growth and exerting antimicrobial activity, avoided the inconveniences caused by the use of the albedo. Moreover, the anti-*Listeria* effectiveness was assessed through a challenge test using a *Listeria* cocktail. The study revealed that *Lactiplantibacillus plantarum* Lpls100, regardless of the presence of the albedo, assures a prompt inhibition of *Listeria* spp. Therefore, its use could be an important contribution to the quality of low-fat fermented sausages.

## 1. Introduction

The consumption of saturated fatty acids (SFAs) and their replacement with unsaturated fatty acids (UFAs) is widely debated [1]. Health organizations all over the world promote the choice of a diet low in saturated fat. The Health Assembly approved, in 2018, the 13th General Programme of Work, which guides the work of the World Health Organization (WHO) in 2019–2023 [2]. Conversely, recent meta-analyses studies found no evidence of a relationship between saturated fat intake and coronary heart disease or cardiovascular disease [3,4]. Despite this evidence, the American Heart Association released a Presidential Advisory on dietary fats with a meta-analysis that provided evidence that re-established their advice to reduce SFAs and replace it with polyunsaturated fatty acids (PUFAs) [5]. Reduction of fat intake and the elimination of industrially-produced *trans*-fats from the food supply are identified in GPW13 as part of WHO’s priority actions to achieve the aims of ensuring healthy lives and promote well-being for all at all ages. In fact, as widely reported in the literature, reducing saturated fat intake causes a potentially important reduction in combined cardiovascular events, and replacing the saturated fat with polyunsaturated fat appears to be a useful strategy [6,7,8,9].

In this context, in the last decade, consumers’ interest in healthy food strongly increased and consequently, the food industry revised the nutrient quality of products, reducing the fat content (mainly saturated fats) and removing unhealthy factors [10]. In particular, low-fat fermented sausages were widely investigated, and several kinds have been introduced by meat industries. However, the individuation of an effective fat replacer requires considerable effort. In fact, fat contributes to the flavour, texture, and overall sensation of the lubricity of meat products [11,12]. Moreover, a reduction of the fat content can affect the acceptability of a product [13]. Therefore, numerous strategies have been developed in order to obtain new formulations of meat products with fat replacements or reductions [14,15]. Dietary fibre, due to its technological and physiological properties, such as compatibility in fat replacement and positive health effects, has been widely investigated [16,17]. Furthermore, products fortified by natural ingredients and by vegetable waste and vegetable by-products could be greatly accepted by consumers, especially by those more sensitive to environmental matters [18]. Fibre-rich by-products, such as albedo produced by *Citrus* spp., have been investigated and discussed for their use as a fat substitute in fermented sausages [19,20]. In particular, it has been highlighted that lemon albedo, due to its high pectin content, increases the water content of fermented sausages, increasing the final yield. Moreover, in a recent review, several positive effects of the albedo on the final quality of fermented sausages were detailed [20]. Lemon albedo use did not affect most sensory properties and promoted juiciness. In addition, lemon albedo, due to its content in bioactive compounds, such as flavonoids and dietary fibre [21,22], could play an important role in the prevention of obesity, atherosclerosis, coronary heart diseases, diabetes, hypercholesterolemia, diverticular disease, and colon cancer [23]. Finally, the majority of the albedo insoluble fibre is fermented in the large intestine, supporting the growth of beneficial intestinal microbiota [21].

However, to date, most of the studies on fat reduction and/or replacement in meat products have mainly focused on the sensory characteristics. In fact, little attention has been paid to the effects of the albedo, due to its ability to retain water and increase the humidity of products. In fact, these effects, some of which are also shown by some authors, could produce a slowdown in the decrease of water activity (a_w_) during the ripening time [16]. Actually, this behavior has a crucial role in Italian fermented sausages and, when a_w_ reduction was slow, food-borne pathogens, such as *Listeria monocytogenes*, were able to survive through to the end of the process. *L. monocytogenes* can be regularly found in raw meat or meat products and is slowly inactivated during the fermentation process [24,25,26]. Hence, in the development of low-fat fermented sausage, using albedo as a fat replacer, additional barriers are required. In the last decade the anti-*Listeria* topic has been investigated, and several studies indicate that the use of protective cultures (Pc) and antagonistic lactic acid bacteria, also with anti-*Listeria* activity, could improve the safety [27,28,29,30,31]. However, even if the achievements in biocontrol strategies applied to Mediterranean-style fermented sausages reached high levels of knowledge [32], the criteria selection generally did not consider the interaction with strains of starter cultures. This aspect is crucial in low-fat fermented sausages which need more of the starter contribution to improve sensory, chemical, and microbiological qualities compared with other conventional fermented sausages. In the light of previous findings and considerations, the present study aimed to determine the microbiological concerns attributable to the use of lemon albedo as a fat replacement and at the development and validation of bioprotective tools compatible with fermented sausages. For this purpose, in the present study, using innovative screening strategies, an appropriate anti-*Listeria* strain was selected, and its effectiveness was verified in situ to assure high quality of low-fat fermented sausages.

## 2. Materials and Methods

### 2.1. Lemon Albedo

Lemon albedo was obtained directly from by-products of a local *Limoncello* (Italian liqueur) manufacturer as reported by Fernandez-Gines et al. [33]. The powder obtained was vacuum packed until use.

### 2.2. Starter Culture

*Latilactobacillus sakei* 152 (*Lt. sakei* 152) and *Staphylococcus xylosus* MVS9 (*S. xylosus* MVS9), both belonging to the Collection of the Department of Agricultural, Environmental, and Food Sciences (DiAAA) of the University of Molise (Italy), were used in this study as a multi-strain starter culture. Both strains were previously isolated from traditional-fermented sausage and selected for their technological features [12,34,35,36].

### 2.3. Protective Microbial Strain Selection

#### 2.3.1. *Lactiplantibacillus plantarum* Producer Strains

Sixty-seven *Lactiplantibacillus plantarum* (*Lb. plantarum*) strains (DiAAA collection, University of Molise) previously isolated from fermented green olives (3), fermented sausage (17), honey bee gut (24), red wines (10), and sourdough (13) were used in the present study [37,38,39,40,41,42,43,44]. The strains, stored at −80 °C [45], were propagated twice in de Man Rogosa Sharpe (MRS, Oxoid, Italy) broth at 28 °C prior to use.

#### 2.3.2. *Listeria* Indicator Strains

*Listeria monocytogenes* (*L. monocytogenes*) ATCC 15313 and four strains of *Listeria innocua* (*L. innocua*), the strain ATCC 33090 and the strains Li03, Li12, Li13, belonging to DIAAA-University of Molise collection [46], were used as indicator strains.

#### 2.3.3. Screening of Anti-*Listeria* Activity of *Lactiplantibacillus plantarum* Strains

The inhibitory effect produced by sixty-seven *Lb. plantarum* strains against *L. monocytogenes* and four strains of *L. innocua* was evaluated. Trials were performed in BHI broth (Oxoid). Growing cells of each indicator strain were inoculated in BHI in order to obtain a final concentration of about 5 Log colony-forming units/mL (CFU/mL) and were combined with cell free supernatant (CFS) of each producer strain. Tubes of BHI without cell free supernatant of producer strains and inoculated with *Listeria* strains as described above, were used as a control. The pH of each tube was adjusted to values of about 5.5 using HCl 1N or NaOH 1N. Tubes were incubated at 30 °C for 30 h because this time corresponds to the advanced stationary phase of *L. innocua* when cultured under control conditions. At the end of the incubation time, 1 mL of sample was withdrawn from each tube, and *Listeria* cells were counted by plate counts performed in BHI agar [28].

The antagonism effect was expressed using an arbitrary index calculated as follows:

$$Anti-Listeria_{score}=-1*\frac{Y_{end\_exp}-Y_{end\_c}}{Y_{end\_c}-Y_{0\_c}}$$

where *Y_end_exp_* represents the count levels of *Listeria* cells after 30 h (t_end_) of incubation in combination with *Lb. plantarum* CFS; *Y_end_c_* represents the count levels of *Listeria* cells after 37 h (t_end_) of incubation under control conditions (without combination); and *Y*_0_*c*_ represents the count levels of *Listeria* cells in the batches at the beginning of the incubation (t_0_) without combination with *Lb. plantarum* CFS. The equation takes into account the effect of producers’ CFS on the final count levels of *L. innocua* $\left(Y_{end\_exp}-Y_{end\_c}\right)$, and normalizing these values with respect to the behaviour under control conditions $\left(Y_{end\_c}-Y_{0_{c}}\right)$, listeria inhibitory and listericidic actions were discerned. In fact, different values of the antimicrobial index indicated diverse anti-*Listeria* effects: *Listeria* inhibitory action (0 < Anti-*Listeria*_score ≤ 1) or Listericidic action (Anti-Listeria_score > 1).

#### 2.3.4. Detection of Interactions between *Lactobacillus plantarum* Strains and Conventional Starter Culture Strains

Sixty-seven *Lb. plantarum* strains were used as producers, while *Lt. sakei* 152 and *S. xylosus* MVS9 were used as indicators. The effect of *Lb. plantarum* strains on the growth of indicators was assessed in sarcoplasmic protein extract (SPE), prepared as described by Basso et al. [47] and inoculated with growing cells of indicator strains combined with cell-free supernatants of producer strains as reported by Tremonte et al. [35]. In all cases, the growing cell inoculum was adjusted to obtain an OD600 nm value of 0.2. The microbial growth of *Lt. sakei* 152 and *S. xylosus* MVS9 after incubation at 28 °C for 30 h was ascertained by plate counts performed in MRS agar and in Mannitol salt agar (MSA), respectively [35,36]. Based on these values, an arbitrary index was estimated and calculated as follows:

$$Indicator\_Compatibility=\frac{Y_{end\_iexp}-Y_{0\_ic}}{Y_{end\_ic}-Y_{0\_ic}}$$

where *Y_end_ic_* and *Y_end_iexp_* represents the count levels of indicators cells at the end of incubation alone or in combination with *Lb. plantarum* CFS, respectively, and *Y*_0_*ic*_ represents the count levels of indicator cells in the batches at the beginning of the incubation (t_0_) without *Lb. plantarum* CFS. Values lower than 0 indicate inhibitory action against the indicators, whereas positive values correspond to a stimulation effect of producers on indicator strains. Values close to zero correspond to a neutral interaction. Specifically, considering previous results [35,36], the following breakpoints were defined:

- High compatibility: Indicator_compatibility ≥ 0.1
- Moderate compatibility: −0.1 < Indicator_compatibility < 0.1
- Low compatibility: Indicator_compatibility ≤ −0.1

### 2.4. Sausage Preparation

#### 2.4.1. Conventional and Low-Fat Fermented Sausage

Salami, southern Italian style, was produced in a small factory by mixing minced pork meat (*longissimus dorsi* and *psoas*) refrigerated at 0 ± 1 °C. During mixing, NaCl (2.5%), glucose (0.3%), black pepper (0.2%), KNO_3_ (0.02%), sterile physiological solution (1% v/w), and the starter culture, containing *Lt. sakei* 152 and *S. xylosus* MVS9 (6 Log CFU/g each), were added. The mixture was divided into four batches: C, control batch produced with minced pork fat addition (14%); A, experimental batch produced with lemon albedo (4%) and without pork fat; CPc, experimental batch produced as C and inoculated with *Lb. plantarum* Lpls100 as a protective culture addition (about 6 Log CFU/g); APc, experimental batch prepared as A but with *Lb. plantarum* Lpls100 as a protective culture addition (about 6 Log CFU/g). After stuffing, the products were left undisturbed for 12 h at about 2–4 °C and were then were dried for seven days (relative humidity increasing from 60 to 80%; temperature decreasing from 18 to 11 °C). Subsequent ripening stages were carried out in store rooms at 80% relative humidity and 11 ± 1 °C temperature for 42 days.

#### 2.4.2. Inoculation of Salami with *Listeria innocua*

In order to simulate *Listeria* contamination during the process, a challenge test on fermented sausage, prepared as reported above, was carried out in parallel. For this purpose, the experimental design proposed by Paratata et al. [48] with some modifications was adopted. In detail, aliquots of each mixture batch described above were used and inoculated (1% v/w) with 3 Log CFU/g of the multi-strain *Listeria* cocktail, a mixture of four strains of *L. innocua*: the strain ATCC 33090 and strains Li03, Li12, and Li13. Prior to use, the microbial cultures were revitalized in BHI (Oxoid, Milan, Italy) and sub-cultured two times in SPE at 18 °C. At the exponential phase, the strains were inoculated in the mixture for the challenge test, obtaining four experimental batches: CL, AL, CPcL, and APcL, which were prepared as the corresponding batches C, A, CPc, and APc, but all inoculated with the multi-strain *Listeria* cocktail.

As reported above, the mixture was stuffed into natural pig casings, and subsequent drying and ripening were carried out. The mixture batches (C, A, CPc, APc) described in the above paragraph were used as control batches. For each treatment (C, A, Cpc, APc, CL, AL, CPcL and APcL), three independent batches were prepared, and two samples from each batch were taken at different time intervals.

### 2.5. Physicochemical, Microbiological, and Sensory Analyses

#### 2.5.1. Physicochemical and Microbiological Analyses

Analyses were performed on samples from each batch at time 0 and after 3, 7, 14, 21, 28, 35, and 42 days of ripening. The pH was determined in three different points of two samples from each batch, using a pH-meter with a Mettler Toledo spear probe (Novate Milanese, Italy). Water activity was measured in triplicate using a Water Activity Meter CR2 (AQUALAB Instrument, Washington, USA) on two different samples from each batch. Microbiological analyses were performed in duplicate on two samples from each batch as reported in the literature [49,50]. Briefly, about 10 g of samples were decimal diluted in a sterile solution of 0.1% peptone water, homogenised in a Stomacher 400 Lab-blender (Seward Medical, London, UK) for 3 min and serially diluted under the same sterile solution. Counts of lactic acid bacteria (LAB), micrococci and staphylococci coagulase- negative cocci (CNC), enterococci, *Brochothrix thermosphacta*, *Listeria* spp., total and faecal coliforms, *Pseudomonas* spp., and *Eumycetes* were detected after appropriate incubation in proper media and conditions as described in a previous study [30,46,50,51]. Results were expressed as the mean of measurements reported as Log CFU/g.

To verify the presence of the starter culture during the ripening period, five colonies were randomly picked up from the MRS and MSA plates. The cultures isolated from MRS (120 cultures) and MSA (150 cultures) agar plates were presumptively identified and typed by RAPD-PCR [12,30,52].

#### 2.5.2. *Listeria* Detection

To detect the behaviour of *Listeria* in the batches intentionally inoculated with the multi-strain *Listeria* cocktail, at time 0 and after 3, 7, 14, 21, 28, 35, and 42 days of ripening, three samples were collected from each batch inoculated with *Listeria* strains (CL; AL; CPcL; and APcL) and from control batches (C, A, CPc, and APc). *Listeria* spp. were enumerated on Oxford agar (Oxoid) prepared with selective supplements (SR140, Oxoid) after incubation at 35 ± 1 °C for 48 ± 2 h. Analyses were performed in duplicate, and the results were expressed as Log CFU/mL.

#### 2.5.3. Fatty-Acid Determination of the Neutral Lipid Fraction

The fatty-acid (FA) compositions were determined by high-resolution gas chromatography, analysing the fatty acid methyl esters obtained by trans-esterification of about 25 mg of each fat sample, which were previously dissolved in 2 mL petroleum ether, and using 2 mL BF_3_-methanol reagent [53]. A Fisons model MFC800 gas chromatograph (Fisons, Milan, Italy) equipped with a 60 m × 0.32 mm i.d. and 0.5 µm film thickness Stabilwax fused silica capillary column (Restek, Bellefonte, PA, USA) was used. The following conditions were applied: (i) oven: 5 min at 170 °C, followed by heating (1 °C/min) up to 220 °C, where the temperature was held for 30 min; (ii) carrier: helium 20 cm/s at 170 °C; (iii) injector: 250 °C, 1 µL; split 40:1; (iv) detector: FID, 250 °C. The FFA composition was determined according to Tremonte et al. [12].

#### 2.5.4. Sensory Analysis

The sensory analysis was performed on salami samples from batches without *Listeria* inoculum (from batches C, A, CPc, and APc) at 42 days of ripening. To define the sensory profile, a descriptive panel of 50 judges, habitual consumers of dry-fermented meat products, was engaged. The judges were trained in preliminary sessions to produce a list of attributes useful to define the sensory profile of low-fat fermented sausage. On the basis of the frequency of citations (>60%), nine descriptors were selected: juiciness, softness, greasiness, rancidity, smell, faecal smell, bitter taste, sour taste, and salty taste. A multiple comparison test was used to determine whether there was a significant difference among the samples from the different batches. A nine-point scale anchored by 1 (extremely low) and 9 (extremely high) was used. Salami samples from each batch were sliced into 2 mm thick slices and equilibrated for 1 h at room temperature according to ISO 8589-1 [54]. Water and unsalted crackers were provided between samples as palate cleansers and to remove any residual flavours.

### 2.6. Statistical Analysis

Statistical analyses were performed following the approach used by Tremonte et al. [40] and by Sorrentino et al. [30]. Similarity in the Anti-*Listeria* profiles was calculated using multivariate analysis. Results obtained from microbiological counts, pH, and a_w_ were analysed by a General Linear Model based on ANOVA considering the effect of batches and ripening time. In all cases, statistical significance was attributed to *p* values of < 0.05. Statistical data were expressed as the mean ± standard error or standard deviation. IBM SPSS Statistics 21 and RStudio (v3.5.0) were used for data analyses and graphical representation.

## 3. Results and Discussion

### 3.1. Anti-Listeria Lactiplantibacillus plantarum Strain Selection

**Figure 1:**
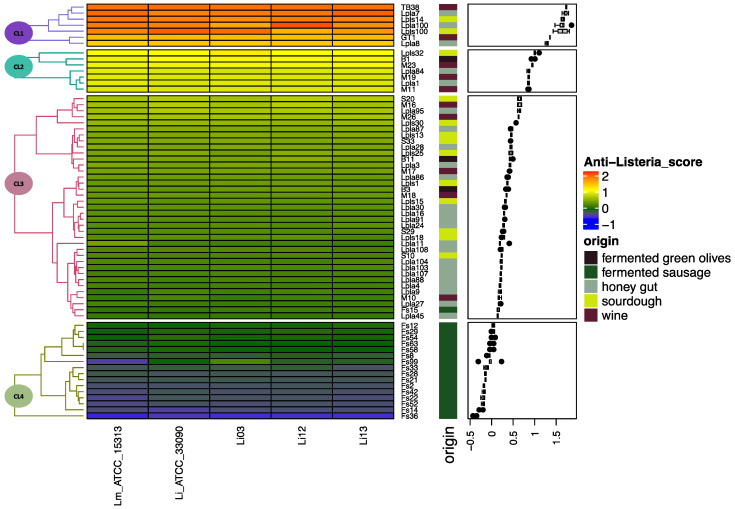
Cluster analysis shown as a heat-map with boxplot annotation concerning the anti-*Listeria* activity (Anti-*Listeria*_score) produced by sixty-seven *Lactiplantibacillus plantarum* strains isolated from different sources (origin). The anti-*Listeria* activity was assayed against *Listeria monocytogenes* (Lm_ATCC_15313) and four strains of *Listeria innocua* (Li_ATCC_33090, Li03, Li12, Li13).

The anti-*Listeria* ability expressed by *Lb. plantarum* strains from diverse isolation sources was investigated. In a previous study, a relationship between the isolation sources characterized by harsh environmental conditions (antimicrobial substances and low pH) and the presence of lactic acid bacteria able to produce a strong antimicrobial activity was highlighted [39,55]. To date, more extensive literature is available on protective or anti-*Listeria* microbial cultures and their advantages and concerns in meat products [27,56]. Selection criteria of protective and starter cultures were also elucidated [57]. However, too little attention has been paid to isolation sources, and the protective strains were isolated and selected only from meat products [58,59,60]. In the present study, *Lb. plantarum* strains from fermented sausages as well as from other and harsher environments, such as wine, sourdough, and honey, were evaluated. Figure 1 shows the heat-map regarding the anti-*Listeria* action expressed by the CFS of sixty-seven *Lb. plantarum* strains against *L. monocytogens* ATCC 15313 and versus the four strains of *L. innocua*. The results are reported as Anti-*Listeria*_score (calculated as reported in Section 2.3.3) assuming values from −1 to +2. Negative values correspond to stimulatory activity of *Listeria* strains, while positive values indicate an inhibition or listericidal action.

The results showed that a response substantially similar was exhibited by the five indicator strains. This finding confirms that *L. innocua* could be considered a surrogate of the pathogen *L. monocytogenes* [61]. On the other hand, significant differences were detected among the producer strains, confirming, as also reported in literature [62], that the antimicrobial activity is a character that is strain-dependent. In particular, the producer strains were grouped into four main clusters (CL1-CL4, Figure 1) depending on their actions against the indicator strains. In detail, sixteen strains, grouped in CL4, did not produce an anti-*Listeria* effect, and eleven of them stimulated *Listeria* growth. It is noteworthy that all strains grouped in CL4 were isolated from fermented sausages. The other assayed strains, all isolated from sources (fermented olives, sourdough, wine, and honeybee guts) characterized by harsher features, exhibited a positive Anti-*Listeria*_score. However, differences in the antagonistic effects were recognized among the producers. In detail, thirty-seven strains (CL3) produced a low-moderate inhibition action, and seven strains (CL2) exhibited a strong inhibitory action. In addition, seven strains (CL1) were distinguished from all others for their listericidal action. In fact, these strains highlighted Anti-*Listeria*_score values higher than 1, indicating a reduction in the *Listeria* count over the incubation time. These last strains were isolated from wine, honeybee guts, and sourdough, all matrices characterized by low pH and other environmental stressors [39,52]. The results confirmed that certain environments with harsh features harbor and select specific strains able to produce antimicrobial activity [39].

#### 3.1.1. Culture Starter Compatibility

**Figure 2:**
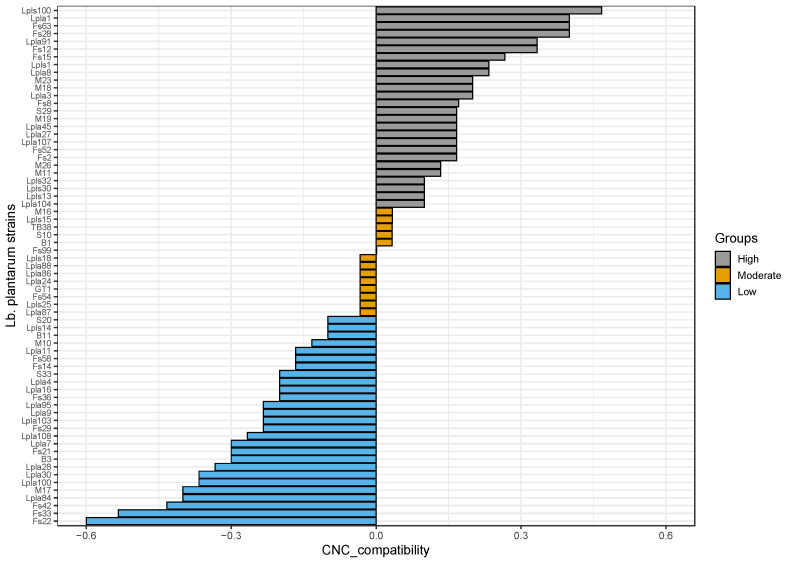
Barplots of the CNC_compatibility index expressed by sixty-seven strains of *Lactiplantibacillus plantarum* against *Staphylococcus xylosus* MVS9. The different colors indicate strains grouped on the basis of high, moderate, or low compatibility.

To evaluate the effect of *Lb. plantarum* strains on the growth starter, growing cells of starter strains (*Lt. sakei* 152 and *S. xylosus* MVS9) were combined with cell-free supernatants of the assayed *Lb. plantarum* strains. By analyzing the values of the “compatibility index”, different situations emerged. In detail, the combination of *Lt. sakei* 152 with the CFS from each *Lb. plantarum* strain showed substantial neutralism, shown by very similar count values between the control (when *Lt. sakei* was cultured singly) and experimental batch (when *Lt. sakei* was cultured in combination). In fact, for all combinations, the values of the “compatibility index”, labelled “sakei compatibility”, were approximately zero (data not shown). Conversely, between *S. xylosus* MVS9 growing cells and *Lb. plantarum* CFSs, several interactions were detected. In fact, as reported in Figure 2, the compatibility index, labelled “CNC compatibility”, showed values ranging about from −0.60 to +0.50.

A moderate interaction was found for 14 combinations between *S. xylosus* MVS9 and the CFS of 14 *Lb. plantarum* strains. Twenty-seven combinations highlighted negative values which indicate the inhibitory action of the related *Lb. plantarum* strains against the indicator. Conversely, twenty-six combinations showed positive values of the CNC compatibility index, denoting a stimulatory action of *Lb. plantarum* strains on the growth of *S. xylosus* MVS9. All these findings emphasized that the choice of a specific *Lb. plantarum* strain, even as a protective culture, could significantly affect the growth of the starter strain. In addition, several previous studies highlighted a strong relationship between the interaction and the expression of important technological activities [12,35,40]. Considering the extensive literature on the CNC role in fermented sausage and particularly on the role of these bacteria in lipolytic, proteolytic, and aromatic behavior [63,64], approaches to promote the growth of these strains should be strongly recommended.

#### 3.1.2. Protective Strain Choice

**Figure 3:**
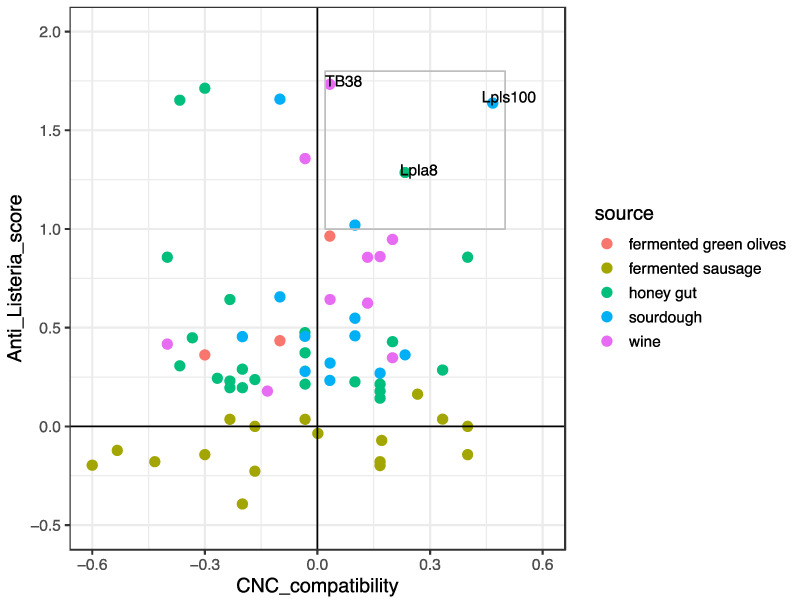
Scatterplot comparing the indexes CNC_compatibility and Anti_*Listeria*_score of sixty-seven strains of *Lactiplantibacillus plantarum* isolated from different sources. The grey box encloses the strains with the two highest indexes.

In order to select the most appropriate protective strain for low-fat and high-quality fermented sausage, the interactions with strains of conventional starter cultures were assessed. This aspect, in the last decade, has been adopted in the selection of fermentative starter cultures [35,36]; however, there is still little consideration of the selection of protective cultures. To date, only Corbo et al. [65], in the selection of antagonistic cultures for sausages, evaluated the interactions with starter strains highlighting substantial neutralism. In our study, several positive or negative interactions between *Lb. plantarum* strains and *S. xylosus* strains were detected. Therefore, data from anti-*Listeria* activity and from the interactions with *S. xylosus* strains were combined and analysed together. In Figure 3, the scatterplot highlights the relationship between “CNC-compatibility” and the “Anti-*Listeria*_score” for each *Lb. plantarum* strain.

On the basis of the results reported in Figure 3, the number of strains useful as protective cultures was strongly reduced. In detail, about half of the strains exhibiting anti-*Listeria* activity also had an inhibitory effect on the growth of *S. xylosus* MVS9. Moreover, only three strains among those with listericidal activities were compatible with the starter strain. In fact, the strains TB38, Lpla8, and Lpls100, isolated from wine, honey guts, and sourdough, respectively, were collocated in the comfortable zone. *Lb. plantarum* Lpls100 was chosen as the highest performing strain and was subsequently used as a protective culture.

### 3.2. Quality Features of Low-Fat Fermented Sausages

In order to evaluate the effects produced by lemon albedo, used as a fat replacement, and by the potential protective effect of *Lb. plantarum* Lpls100, conventional and experimental batches of fermented sausages were prepared and monitored during ripening in order to investigate the principal quality features. Moreover, the presence of the bacterial strains used as starter strains or protective strains was ascertained. For this purpose, typing was performed by RAPD-PCR on both lactobacilli and CNC isolated from all batches (C, A, CPc, APc, CL, AL, CPcL, and APcL) at 0, 21, and 42 days of ripening. The results highlighted the effectiveness of the inoculation and sustained ability of the strains used as starters to survive during the ripening of salami. In fact, more than 90% of presumptive CNC isolates from the four batches had RAPD-PCR profiles with a coefficient of similarity higher than 85% with *S. xylosus* MVS9. Similar results were obtained for lactobacilli isolated from MRS plates; in fact, about 90% of isolates, phenotypically identified as *Lt. sakei* and *Lb. plantarum*, showed a coefficient of similarity higher than 85% with the strains *Lt. sakei* 152 and *Lb. plantarum* Lpls100, used as starters and protective culture (data not shown).

#### 3.2.1. Physicochemical Features of Conventional and Low-Fat Sausages

**Figure 4:**
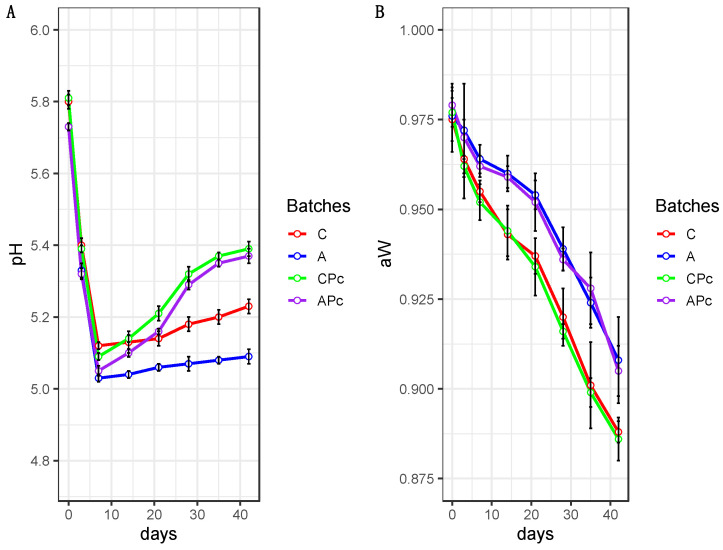
Line plots showing the behaviour of pH (**A**) and a_w_ (**B**) in samples from batch C, conventional fermented sausage prepared with starter culture and 14% minced pork fat addition; batch A, prepared with 4% lemon albedo in total replacement of pork fat and starter culture addition; batch CPc, prepared with starter culture and 14% minced pork addition plus anti-*Listeria Lactiplantibacillus plantarum* Lpls100; batch APc, prepared with 4% lemon albedo in total replacement of pork fat and starter culture addition plus anti-*Listeria Lactiplantibacillus plantarum* Lpls100. The data are reported as mean values with error bars.

The behaviours of pH and a_w_ of each batch, during ripening, are shown in Figure 4A,B respectively. The pH values resulted in agreement with those commonly found in other fermented sausages from southern Italy [40,66,67]. However, ripening time and batches affected pH values (*p* < 0.05). In all batches, a significant decrease in pH values was detected during the ripening time, falling from 5.80 ± 0.09 to values ranging between 5.03 ± 0.02 and 5.12 ± 0.02 after 7 days of ripening. This behaviour was consistent with those revealed in Italian sausage prepared with starter cultures [68,69,70]. This last finding suggests the effectiveness of the starter. As regard the different batches, during the entire period time, in the samples from batch A were detected pH values lower than those observed in the other batches. Also, other authors found that the use of citrus fibre at 4% produced a decrease in the pH values [16,71]. Moreover, in the samples from batch A, the pH values decrease until the 7th day, a behaviour substantially constant were detecting. A similar trend, also with values slightly higher, characterized also the samples from batch C.

Conversely, the samples from batches CPc and APc highlighted a significant increase from the 14th day of ripening. Moreover, in these samples, during the entire ripening period, similar values were detected. Therefore, the presence of the anti-*Listeria* strain would seem to remove the effect, even if slight, due to albedo. However, this aspect deserves further attention, also considering the behaviour in microbial populations. In detail, the protective strain *Lb. plantarum* Lpls100, due to its ability to promote the growth of the proteolytic strain *S. xylosus* MVS9, could produce an increase in pH values affecting the sensory features of the product.

The a_w_ (Figure 4B) decreased from initial values of about 0.97 to about 0.89 at the end of ripening. However, regardless of the presence of *Lb. plantarum* Lpls100, significant differences emerged among the batches with or without albedo. In fact, the a_w_ recorded in A and APc from the 14th day of ripening was about 0.02 units higher than that recorded in relative batches without albedo (batches C and CPc). These findings are in agreement with the literature [16] and could be attributable to the properties of lemon albedo that, due to its high pectin content (25%), produces a water content increase in the product [20,72]. All these properties, in our opinion, could also affect other quality features, such as microbiological and sensory properties. In fact, the lower decrease in a_w_ values, as also evidenced in a recent study [48], could affect the microbial behaviour, compromising the more prompt inhibition of undesirable microbial groups.

#### 3.2.2. Microbiological Features of Conventional and Low-Fat sausages

**Figure 5:**
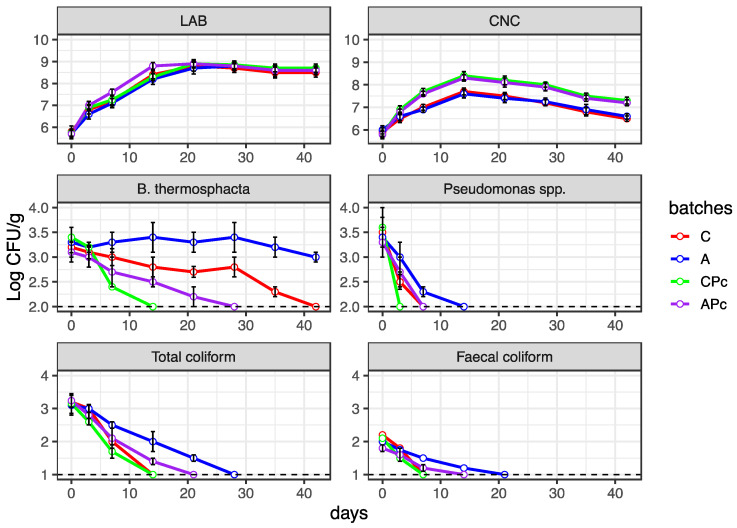
Line plots showing the behaviours of the main microbial groups in samples from batch C, conventional fermented sausage prepared with starter culture and 14% minced pork fat additions; batch A, prepared with 4% lemon albedo in total replacement of pork fat and starter culture addition; batch CPc, prepared with starter culture and 14% minced pork addition plus anti-*Listeria Lactiplantibacillus plantarum* Lpls100; batch APc, prepared with 4% lemon albedo in total replacement of pork fat and starter culture addition plus anti-*Listeria Lactiplantibacillus plantarum* Lpls100. The data are reported as the mean value with error bars. The dashed lines represent the limit of detection of the microbial groups.

The behaviour of the most relevant microorganisms is reported in Figure 5. Except for yeast and moulds which showed a substantially constant trend during ripening (data not shown), for all microbial groups, significant differences (*p* < 0.05) in microbial load were found depending on the different sampling time and the diverse batches. During the ripening period, a significant increase in LAB and CNC count levels was observed, whereas a decrease in levels of undesirable microbial populations was detected. This is consistent with literature highlighting, once again, that useful lactic acid bacteria and CNC effectively contribute to the ripening process [73,74,75]. Moreover, data also showed that diverse spoilage bacteria, such as *Brochothrix thermosphacta*, *Pseudomonas* spp., and Coliforms persisted and were detectable until the advanced stage of ripening. These microorganisms are widely described as contaminants of fresh meat as well as the most common spoilage microorganisms of fermented meat products [76,77,78,79].

However, several differences were found among the different batches. For LAB counts, similar count levels were detected in all batches. This finding indicates that the use of the albedo did not affect the growth of useful strain starters. Interesting differences among the batches were noticed in CNC behaviour. Specifically, even if the same trend was detected in all batches, the samples prepared with protective culture (APc and CPc) addition showed count levels higher than those observed in samples from batches A and C. This difference could be related to the ability of selected *Lb. plantarum* Lpls100 to promote the growth of *S. xylosus* MVS9. Moreover, the higher levels of CNC would also justify the pH increase during the second step of ripening, which was higher in APc and CPc than in batches A and C. In fact, as already reported in a previous study, CNC plays a significant role in proteolytic activity and consequently an increase in pH values [35,36]. Particular attention should be paid to the differences among the batches concerning the undesirable microorganisms. A greater persistence of certain *Enterobacteriaceae* (total and faecal coliforms), of *Pseudomonas* spp. as well as of *Brochothrix thermosphacta* was found in the samples from batch A. In particular, *B. thermosphacta* was detected in batch A until the end of ripening. This bacterium belonging to the Listeriaceae family, is phylogenetically related to *L. monocytogenes,* and is considered a strict meat spoiler [80]. A similar situation was also detected for *Listeria* spp. (data not shown) which, even if found since time 0 of ripening at negligible values, persisted in batch A until the end of ripening. The greater persistence of undesirable bacteria in sample from batch A could be related to their a_w_ values being higher than those detected in the samples from batches C and CPc. However, for the samples from batch APc, even if characterized by a_w_ values similar to those detected in batch A, the prompt decrease in undesirable bacteria was assured by the presence of protective *Lb. plantarum* Lpls100. In detail, the effect of Lpls100 on *Listeria* spp. was elucidated in Section 3.3.

#### 3.2.3. Final Fatty Acid Composition and Sensory Features

**Figure 6:**
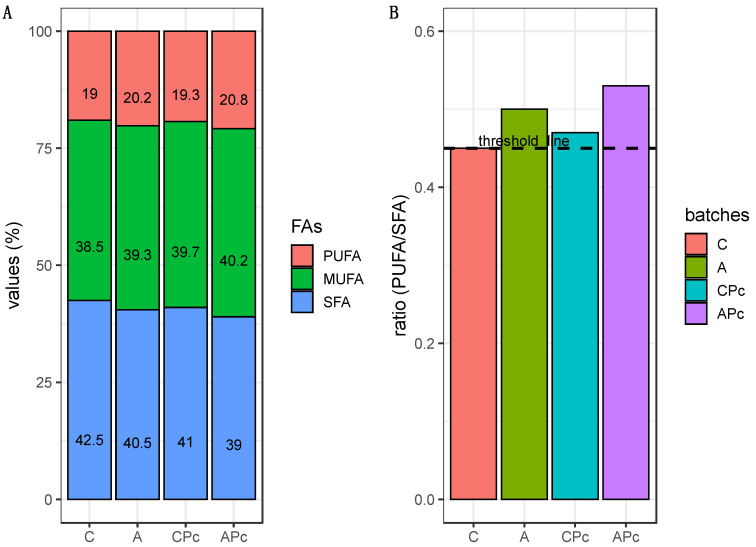
Stacked bar plot (**A**) showing the distribution of fatty acids (PUFA, MUFA, SFA) and bar plot (**B**) showing the ratio PUFA/SFA in samples from batch C, conventional fermented sausage prepared with starter culture and 14% minced pork fat additions; batch A, prepared with 4% lemon albedo in total replacement of pork fat and starter culture addition; batch CPc, prepared with starter culture and 14% minced pork addition plus anti-*Listeria Lactiplantibacillus plantarum* Lpls100; batch APc, prepared with 4% lemon albedo in total replacement of pork fat and starter culture addition plus anti-*Listeria Lactiplantibacillus plantarum* Lpls100. The dashed line represents the threshold value 0.45.

Fatty acids (FAs) represent an important component in fermented sausages, affecting their sensory and nutritional features [40,81]. The percentages of unsaturated fractions and of saturated fatty acids are reported in Figure 6A. Results showed that unsaturated fatty acids (MUFA + PUFA) were higher than saturated fatty acids (SFA) in all batches. This finding is consistent with other studies reported in the literature [82,83]. However, as highlighted in Figure 6A, differences among the batches were noticed. In particular, considering values of PUFA and SFA levels, significant differences among the batches could be revealed. For this purpose, the PUFA/SFA ratio was considered (Figure 6B). This ratio has been adopted as an index to describe the quality of fermented sausages, and the nutritional recommendations highlighted that the PFA/SFA ratio should be higher than 0.45 [84]. Interesting results were shown by the samples from all experimental batches. In detail, ratio values close to the threshold of 0.45 were exhibited by the samples from batch C. All others samples highlighted PUFA/SFA ratios significantly higher than 0.45. Specifically, a higher ratio value (0.53) was shown by the samples from batch APc. Therefore, on the basis of this last evidence, a role of both albedo and protective strain Lpls100 could be assumed.

**Figure 7:**
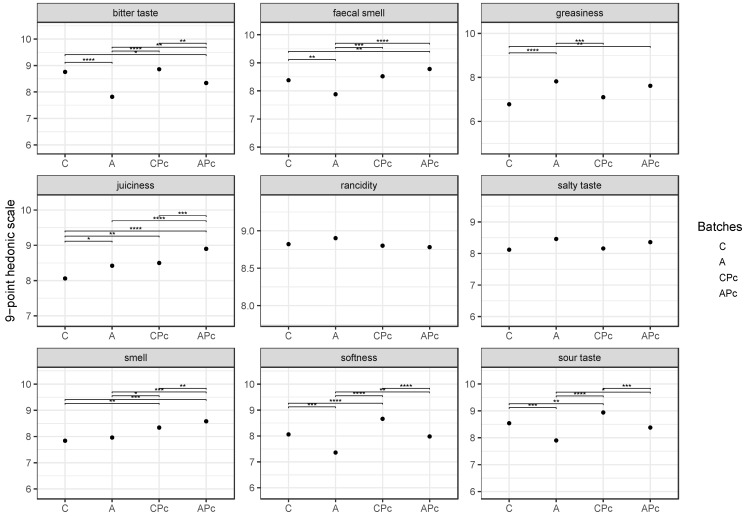
Jitter plot showing nine sensory attributes at the end of ripening time (42 days) in samples from batch C, conventional fermented sausage prepared with starter culture and 14% minced pork fat additions; batch A, prepared with 4% lemon albedo in total replacement of pork fat and starter culture addition; batch CPc, prepared with starter culture and 14% minced pork addition plus anti-*Listeria Lactiplantibacillus plantarum* Lpls100; batch APc, prepared with 4% lemon albedo in total replacement of pork fat and starter culture addition plus anti-*Listeria Lactiplantibacillus plantarum* Lpls100. Black dots represent the mean value of 50 records. Statistical significance: **** *p* < 0.0001; *** *p* < 0.001; ** *p* < 0.01; * *p* < 0.05.

In addition to the nutritional aspects, the fat replacement could affect the sensory features since fats have an important role in appearance, softness, flavour, smell, juiciness, and rancidity. As reported in the literature, the addition of fat substitutes in fermented sausages influences the final texture [85]. However, to date, the effect of lemon albedo is still debated [20]. Some authors showed that the addition of lemon albedo to fermented sausages did not affect sensory properties such as smell, or salty and acidic taste, and increased juiciness [19], while others assured that the addition of citrus fibre in fermented sausages decreased the smell, consistency, and juiciness [33]. On these bases, the effect of lemon albedo and of protective strain Lpls100 on sensory attributes was also ascertained by a panel of trained judges [54]. The mean values of sensory attributes are reported in Figure 7. The results showed that fat replacement and protective strain Lpls100 did not negatively affect the sensory features and they positively influenced some attributes. In detail, no significant differences among the batches with albedo lemon (A, APc) or without (C and CPc) were found in juiciness, salty taste, and rancidity. This last finding is interesting considering that so far the rancidity and the lipid oxidation represent the main critical issues in the preparation of fermented sausage. The samples from batch A showed lower softness, faecal smell, bitter and sour taste than those from the other batches. These attributes and in particular the faecal smell could be due to the persistence of several spoilage microorganisms in samples prepared with albedo addition (batch A). However, the combination of the albedo with protective strains (APc) enhanced the sensory quality, preventing the low softness, bitter, and sour taste perception. This finding could be due to the antimicrobial effect of protective stain Lpls100 as well as to its ability to promote CNC growth that, as reported in previous studies, could positively contribute to the texture and sensory features [12,35].

### 3.3. Anti-Listeria Action in Low-Fat Fermented Sausages

Based on the results reported in the previous paragraph, it clearly appeared that the main concern of low-fat fermented sausages is the microbiological quality. In this context, the evaluation of the effect expressed by the albedo and the protective strain *Lb. plantarum* Lpls100 on the behaviour of a multi-strain *Listeria* cocktail, intentionally added, could offer useful knowledge. For this purpose, four experimental batches (CL, AL, CPcL, and APcL) were prepared as the corresponding batches C, A, CPc, and APc and inoculated with the multi-strain *Listeria* cocktail.

**Figure 8:**
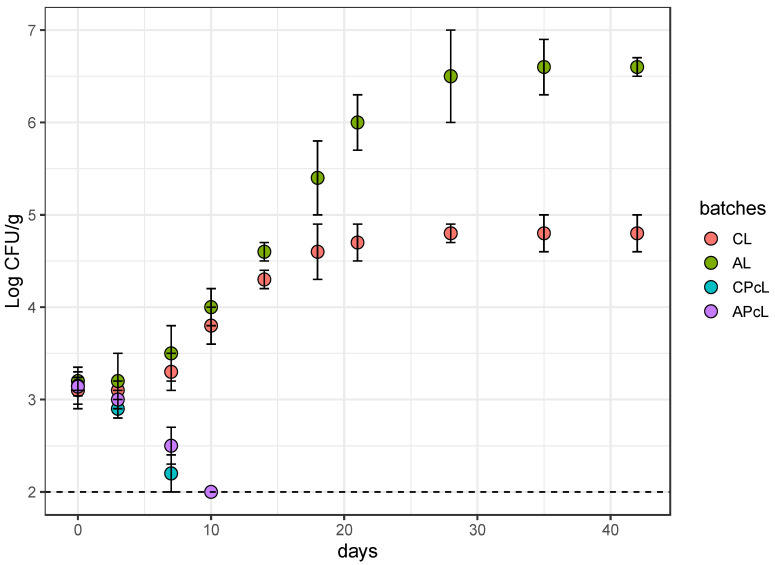
Scatterplot showing the challenge test of *Listeria* spp. during the ripening of fermented sausage from batch CL, fermented sausage prepared with starter culture and 14% minced pork Figure 4. strains of *L. innocua* (3 Log CFU/g); batch AL, prepared with 4% lemon albedo in total replacement of pork fat and starter culture addition 4 strains of *L. innocua* (3 Log CFU/g); batch CPcL, prepared with starter culture and 14% minced pork addition plus anti-*Listeria Lactiplantibacillus plantarum* Lpls100 and 4 strains of *L. innocua* (3 Log CFU/g); batch APcL, prepared with 4% lemon albedo in total replacement of pork fat and starter culture addition plus anti-*Listeria Lactiplantibacillus plantarum* Lpls100 and 4 strains of *L. innocua* (3 Log CFU/g).

During the ripening time, the samples from the batches inoculated with the *Listeria* cocktail showed values of pH and a_w_ closely comparable to those detected in the samples from the relative control batches (C, A, CPc, and APc)—data not shown. Specifically, the samples from the batches AL and APcL highlighted a_w_ values about 0.02 units higher than those detected in the samples from batches CL and CPcL. Moreover, for the pH values, differences among the batches were detected during the second step of ripening. In detail, in the samples from batches CPcL and APcL, higher pH values were detected than those registered in samples from CL and AL. Moreover, the effective presence of the strains used as starter or protective culture was determined by RAPD-PCR and as reported above, their occurrences were confirmed at 0, 21, and 42 days of ripening. Therefore, the starter and protective strains represented the main dominant microbial population during the fermentative process. The effectiveness on the protective strain Lpls100 was clearly shown by Figure 8, which reports the behaviours of *Listeria* spp. in the different batches. Specifically, the behaviours of *Listeria* spp. in the four batches showed two different trends.

In the batches without protective strain addition, an increase from 3.2 Log CFU/g to 4.9 Log CFU/g (batch CL) or 6.6 Log CFU/g (batch AL) was detected. Conversely, for the batches prepared with protective culture addition, a decrease was highlighted. This last evidence clearly highlights the protective and anti-*Listeria* effectiveness of *Lb. plantarum* Lpls100. However, to better understand the role of the albedo, the significant differences between batches AL and CL in the increasing behaviour deserve to be considered. Specifically, in these batches, the *Listeria* count levels appeared substantially similar until the 18th day of ripening when significant differences were observed, and, in samples from batch AL, count levels almost 2 Log CFU/g higher than those revealed in the samples from batch CL were observed. Therefore, the albedo and the related environmental features, such as physicochemical parameters, promote the growth of *Listeria* spp. In detail, based on our results and considering the literature, the a_w_ parameter seems to be crucial in the behaviour of *Listeria* spp. [86]. These assertions are consistent with other evidence in the literature reporting that at fermentation temperatures below or equal to 20 °C, a_w_ reduction became more important than pH decreases and if this reduction is slow, *Listeria* may survive longer [87]. Moreover, other authors showed that although the pH decrease was rapid during the first 48 h of fermentation, the slow dehydration of the sausages and the small change of a_w_ did not contribute to rapid inactivation of the pathogens.

## 4. Conclusions

Lemon albedo could represent a suitable substitute for total replacement of pork fat in the preparation of fermented sausages. However, for its use to be feasible, careful consideration is needed. In fact, on the one hand, the albedo did not affect the behaviour of useful fermentative bacteria, such as *lactic acid bacteria* and CNC, and positively influenced nutritional features and some sensory attributes such as juiciness, greasiness, and salty taste. On the other hand, because of its ability to retain water, the albedo produced a slow decrease in a_w_ values. Actually, the a_w_ decrease seems to be crucial for the behaviour of *Listeria* that harbours the pathogen species *L. monocytogenes*, as well as for the behaviour of spoilage bacteria, such as *B. thermosphacta*. Considering the significant *Listeria* risk for fermented sausage, the use of additional and bio-protective barriers in the production of low-fat fermented sausages is essential. Moreover, an appropriate selection of protective culture is fundamental. In fact, the antagonistic action against the undesirable bacterial target as well as the synergic or compatible relationship with starter strains must be considered. *Lb plantarum* Lpls100, highlighting high compatibility with starter strains and a strong anti-*Listeria* action, could be effectively used to assure the high microbiological quality of low-fat fermented sausages.

## References

[1] Heileson J.L. (2020). Dietary saturated fat and heart disease: A narrative review. Nutr. Rev..

[2] World Health Organization (2018). Thirteenth General Programme of Work (GPW13) 2019–2023.

[3] Siri-Tarino P.W., Sun Q., Hu F.B., Krauss R.M. (2010). Meta-analysis of prospective cohort studies evaluating the association of saturated fat with cardiovascular disease. Am. J. Clin. Nutr..

[4] Ramsden C.E., Zamora D., Leelarthaepin B., Majchrzak-Hong S.F., Faurot K.R., Suchindran C.M., Ringel A., Davis J.M., Hibbeln J.R.. (2013). Use of dietary linoleic acid for secondary prevention of coronary heart disease and death: Evaluation of recovered data from the Sydney Diet Heart Study and updated meta-analysis. BMJ.

[5] Sacks F.M., Lichtenstein A.H., Wu J.H., Appel L.J., Creager M.A., Kris-Etherton P.M., Miller M., Rimm E.B., Rudel L.L., Robinson J.G. (2017). Dietary fats and cardiovascular disease: A presidential advisory from the American Heart Association. Circulation.

[6] Hooper L., Martin N., Jimoh O.F., Kirk C., Foster E., Abdelhamid A.S. (2020). Reduction in saturated fat intake for cardiovascular disease. Cochrane Database Syst. Rev..

[7] WHO (2003). Diet, Nutrition and the Prevention of Chronic Diseases: Report of a Joint WHO/FAO Expert Consultation.

[8] FAO (2010). Fats and Fatty Acids in Human Nutrition: Report of an Expert Consultation.

[9] Cengiz E., Gokoglu N. (2005). Changes in energy and cholesterol contents of frankfurter-type sausages with fat reduction and fat replacer addition. Food Chem..

[10] Vlassopoulos A., Masset G., Leroy F., Hoover C., Chesneau-Guillemont C., Leroy F., Lehmann U., Spieldenner J., Tee E.-S., Gibney M. (2017). A nutrient profiling system for the (re)formulation of a global food & beverage portfolio. Eur. J. Nutr..

[11] Mendoza E., García M.L., Casas C., Seglas M.D. (2001). Inulin as fat substitute in low fat, dry fermented sausages. Meat Sci..

[12] Tremonte P., Gambacorta G., Pannella G., Trani A., Succi M., La Gatta B., Tipaldi L., Grazia L., Sorrentino E., Coppola R. (2018). NaCl replacement with KCl affects lipolysis, microbiological and sensorial features of *soppressata* molisana. Eur. J. Lipid Sci. Technol..

[13] Samapundo S., Xhaferi R., Szczcepaniak S., Goemare O., Steen L., Paelinck H., Devlieghere F. (2015). The effect of water soluble fat replacers and fat reduction on the growth of *Lactobacillus sakei* and *Listeria monocytogenes* in broth and pork liver paté. LWT-Food Sci. Technol..

[14] Muguerza E., Gimeno O., Ansorena D., Astiasarán I. (2004). New formulations for healthier dry fermented sausages: A review. Trends Food Sci. Technol..

[15] Jiménez-Colmenero F., Salcedo-Sandoval L., Bou R., Cofrades S., Herrero A.M., Ruiz-Capillas C. (2015). Novel applications of oil-structuring methods as a strategy to improve the fat content of meat products. Trends Food Sci. Technol..

[16] Yalınkılıç B., Kaban G., Kaya M. (2012). The effects of different levels of orange fiber and fat on microbiological, physical, chemical and sensorial properties of sucuk. Food Microbiol..

[17] Fernandez-Lopez J., Sendra E., Sayas-Barbera E., Navarro C., Perez-Alvarez J.A. (2008). Physico-chemical and microbiological profiles of “salchichon” (Spanish dry-fermented sausage) enriched with orange fiber. Meat Sci..

[18] Kowalska H., Czajkowska K., Cichowska J., Lenart A. (2017). What’s new in biopotential of fruits and vegetable by-products applied in the food processing industry. Trends Food Sci. Technol..

[19] Aleson-Carbonell L., Fernández-López J., Pérez-Alvarez J.A., Kuri V. (2005). Functional and sensory effects of fibre-rich ingredients on breakfast fresh sausages manufacture. Food Sci. Technol. Int..

[20] Calderón-Oliver M., López-Hernández L.H. (2020). Food vegetable and fruit waste used in meat products. Food Rev. Int..

[21] Caggia C., Palmeri R., Russo N., Timpone R., Randazzo C.L., Todaro A., Barbagallo S. (2020). Employ of citrus by-product as fat replacer ingredient for bakery confectionery products. Front. Nutr..

[22] Mirabella N., Castellani V., Sala S. (2014). Current options for the valorization of food manufacturing waste: A review. J. Clean. Prod..

[23] Dhingra D., Michael M., Rajput H., Patil R.T. (2011). Dietary fiber in foods: A review. J. Food Sci. Technol..

[24] Lücke F.K. (2000). Utilization of microbes to process and preserve meat. Meat Sci..

[25] Iacumin L., Manzano M., Comi G. (2016). Phage inactivation of *Listeria monocytogenes* on San Daniele dry-cured ham and elimination of biofilms from equipment and working environments. Microorganisms.

[26] Comi G., Cocolin L., Cantoni C., Manzano M. (1997). A RE-PCR method to distinguish *Listeria monocytogenes* serovars. FEMS Immunol. Med. Microbiol..

[27] Comi G., Andyanto D., Manzano M., Iacumin L. (2016). *Lactococcus lactis* and *Lactobacillus sakei* as bio-protective culture to eliminate *Leuconostoc mesenteroides* spoilage and improve the shelf life and sensorial characteristics of commercial cooked bacon. Food Microbiol..

[28] Tremonte P., Succi M., Coppola R., Sorrentino E., Tipaldi L., Picariello G., Pannella G., Fraternali F. (2016). Homology-based modeling of universal stress protein from *Listeria innocua* up-regulated under acid stress conditions. Front. Microbiol..

[29] Giello M., La Storia A., De Filippis F., Ercolini D., Villani F. (2018). Impact of *Lactobacillus curvatus* 54M16 on microbiota composition and growth of *Listeria monocytogenes* in fermented sausages. Food Microbiol..

[30] Sorrentino E., Succi M., Tipaldi L., Pannella G., Maiuro L., Sturchio M., Coppola R., Tremonte P. (2018). Antimicrobial activity of gallic acid against food-related *Pseudomonas* strains and its use as biocontrol tool to improve the shelf life of fresh black truffles. Int. J. Food Microbiol..

[31] Abdulhussain Kareem R., Razavi S.H. (2020). Plantaricin bacteriocins: As safe alternative antimicrobial peptides in food preservation—A review. J. Food Safety.

[32] Oliveira M., Ferreira V., Magalhães R., Teixeira P. (2018). Biocontrol strategies for Mediterranean-style fermented sausages. Food Res. Int..

[33] Fernandez-Gines J.M., Fernandez-Lopez J., Sayas-Barbera E., Sendra E., Perez- Alvarez J.A. (2003). Effect of storage conditions on quality characteristics of Bologna sausages made with citrus fiber. J. Food Sci..

[34] Di Luccia A., Tremonte P., Trani A., Loizzo P., La Gatta B., Succi M., Sorrentino E., Coppola R. (2016). Influence of starter cultures and KCl on some biochemical, microbiological and sensory features of soppressata molisana, an Italian fermented sausage. Eur. Food Res. Technol..

[35] Tremonte P., Reale A., Di Renzo T., Tipaldi L., Di Luccia A., Coppola R., Sorrentino E., Succi M. (2010). Interactions between *Lactobacillus sakei* and CNC (*Staphylococcus xylosus* and *Kocuria varians*) and their influence on proteolytic activity. Lett. Appl. Microbiol..

[36] Tremonte P., Succi M., Reale A., Di Renzo T., Sorrentino E., Coppola R. (2007). Interactions between strains of *Staphylococcus xylosus* and *Kocuria varians* isolated from fermented meats. J. Appl. Microbiol..

[37] Lombardi S.J., Pannella G., Iorizzo M., Testa B., Succi M., Tremonte P., Sorrentino E., Di Renzo M., Strollo D., Coppola R. (2020). Inoculum strategies and performances of malolactic starter *Lactobacillus plantarum* M10: Impact on chemical and sensorial characteristics of Fiano Wine. Microorganisms.

[38] Succi M., Pannella G., Tremonte P., Tipaldi L., Coppola R., Iorizzo M., Lombardi S.J., Sorrentino E. (2017). Sub-optimal pH preadaptation improves the survival of *Lactobacillus plantarum* strains and the malic acid consumption in wine-like medium. Front. Microbiol..

[39] Tremonte P., Pannella G., Succi M., Tipaldi L., Sturchio M., Coppola R., Luongo D., Sorrentino E. (2017). Antimicrobial activity of *Lactobacillus plantarum* strains isolated from different environments: A preliminary study. Int. Food Res. J..

[40] Tremonte P., Sorrentino E., Pannella G., Tipaldi L., Sturchio M., Masucci A., Maiuro L., Coppola R., Succi M. (2017). Detection of different microenvironments and *Lactobacillus sakei* biotypes in Ventricina, a traditional fermented sausage from central Italy. Int. J. Food Microbiol..

[41] De Leonardis A., Testa B., Macciola V., Lombardi S.J., Iorizzo M. (2016). Exploring enzyme and microbial technology for the preparation of green table olives. Eur. Food Res. Technol..

[42] Iorizzo M., Lombardi S.J., Macciola V., Testa B., Lustrato G., Lopez F., De Leonardis A. (2016). Technological potential of *Lactobacillus* strains isolated from fermented green olives: In vitro studies with emphasis on oleuropein-degrading capability. Sci. World J..

[43] Iorizzo M., Testa B., Lombardi S.J., García-Ruiz A., Muñoz-González C., Bartolomé B., Moreno-Arribas M.V. (2016). Selection and technological potential of *Lactobacillus plantarum* bacteria suitable for wine malolactic fermentation and grape aroma release. LWT.

[44] Sorrentino E., Reale A., Tremonte P., Maiuro L., Succi M., Tipaldi L., Renzo T., Pannella G., Coppola R. (2013). *Lactobacillus plantarum* 29 inhibits *Penicillium* spp. involved in the spoilage of black truffles (*Tuber aestivum*). J. Food Sci..

[45] Succi M., Tremonte P., Reale A., Sorrentino E., Coppola R. (2007). Preservation by freezing of potentially probiotic strains of *Lactobacillus rhamnosus*. Ann. Microbiol..

[46] Pannella G., Messia M.C., Tremonte P., Tipaldi L., La Gatta B., Lombardi S.J., Succi M., Marconi E., Coppola R., Sorrentino E. (2019). Concerns and solutions for raw milk from vending machines. J. Food Process. Pres..

[47] Basso A.L., Picariello G., Coppola R., Tremonte P., Spagnamusso S., Di Luccia A. (2004). Proteolytic activity of *Lactobacillus sakei*, *Lactobacillus farciminis* and *Lactobacillus plantarum* on sarcoplasmic proteins of pork lean. J. Food Biochem..

[48] Patarata L., Novais M., Fraqueza M.J., Silva J.A. (2020). Influence of Meat Spoilage Microbiota Initial Load on the Growth and Survival of Three Pathogens on a Naturally Fermented Sausage. Foods.

[49] Tremonte P., Sorrentino E., Succi M., Tipaldi L., Pannella G., Ibañez E., Mendiola J.A., Di Renzo T., Reale A., Coppola R. (2016). Antimicrobial effect of *Malpighia punicifolia* and extension of water buffalo steak shelf-life. J. Food Sci..

[50] Tremonte P., Sorrentino E., Succi M., Reale A., Maiorano G., Coppola R. (2005). Shelf life of fresh sausages stored under modified atmospheres. J. Food Prot..

[51] Testa B., Lombardi S.J., Macciola E., Succi M., Tremonte P., Iorizzo M. (2019). Efficacy of olive leaf extract (*Olea europaea* L. cv Gentile di Larino) in marinated anchovies (*Engraulis encrasicolus*, L.) process. Heliyon.

[52] Testa B., Lombardi S.J., Tremonte P., Succi M., Tipaldi L., Pannella G., Sorrentino E., Iorizzo M., Coppola R. (2014). Biodiversity of *Lactobacillus plantarum* from traditional Italian wines. World J. Microbiol. Biotechnol..

[53] Folch J., Lees M., Sloane-Stanley G.H. (1957). A simple method for the isolation and purification of total lipids from animal tissues. J. Biol. Chem..

[54] International Organization for Standardization (1998). ISO 8589: Sensory Analysis—General Guidance for the Design of Test Rooms.

[55] Iorizzo M., Lombardi S.J., Ganassi S., Testa B., Ianiro M., Letizia F., Succi M., Tremonte P., Vergalito F., Cozzolino A. (2020). Antagonistic Activity against *Ascosphaera apis* and Functional Properties of *Lactobacillus kunkeei* Strains. Antibiotics.

[56] Vinicius De Melo Pereira G., De Carvalho Neto D.P., Junqueira A.C.D.O., Karp S.G., Letti L.A., Magalhães Júnior A.I., Soccol C.R. (2020). A review of selection criteria for starter culture development in the food fermentation industry. Food Rev. Int..

[57] Laranjo M., Potes M.E., Elias M. (2019). Role of starter cultures on the safety of fermented meat products. Front. Microbiol..

[58] Zagorec M., Champomier-Vergès M.C. (2017). *Lactobacillus sakei*: A starter for sausage fermentation, a protective culture for meat products. Microorganisms.

[59] Biscola V., Todorov S.D., Capuano V.S.C., Abriouel H., Gálvez A., Franco B.D. (2013). Isolation and characterization of a nisin-like bacteriocin produced by a *Lactococcus lactis* strain isolated from charqui, a Brazilian fermented, salted and dried meat product. Meat Sci..

[60] de Souza Barbosa M., Todorov S.D., Ivanova I., Chobert J.M., Haertlé T., De Melo Franco B.D.G. (2015). Improving safety of salami by application of bacteriocins produced by an autochthonous *Lactobacillus curvatus* isolate. Food Microbiol..

[61] Girardin H., Morris C.E., Albagnac C., Dreux N., Glaux C., Nguyen-The C. (2005). Behaviour of the pathogen surrogates *Listeria innocua* and *Clostridium sporogenes* during production of parsley in fields fertilized with contaminated amendments. FEMS Microbiol. Ecol..

[62] Castellano P., Pérez Ibarreche M., Blanco Massani M., Fontana C., Vignolo G.M. (2017). Strategies for pathogen biocontrol using lactic acid bacteria and their metabolites: A focus on meat ecosystems and industrial environments. Microorganisms.

[63] Iacumin L., Osualdini M., Bovolenta S., Boscolo D., Chiesa L., Panseri S., Comi G. (2020). Microbial, chemico-physical and volatile aromatic compounds characterization of Pitina PGI, a peculiar sausage-like product of North East Italy. Meat Sci..

[64] Mainar M.S., Stavropoulou D.A., Leroy F. (2017). Exploring the metabolic heterogeneity of coagulase-negative staphylococci to improve the quality and safety of fermented meats: A review. Int. J. Food Microbiol..

[65] Corbo M.R., Bevilacqua A., Speranza B., Gallo M., Campaniello D., Sinigaglia M. (2017). Selection of wild lactic acid bacteria for sausages: Design of a selection protocol combining statistic tools, technological and functional properties. LWT-Food Sci. Technol..

[66] Sorrentino E., Tremonte P., Capobianco F., Succi M., Reale A., Di Renzo T., Coppola R. (2007). Rapporti di interazione tra microrganismi di interesse tecnologico isolati da soppressata molisana. Ind. Aliment..

[67] Urso R., Comi G., Cocolin L. (2006). Ecology of lactic acid bacteria in Italian fermented sausages: Isolation, identification and molecular characterization. Syst. Appl. Microbiol..

[68] Cardinali F., Milanović V., Osimani A., Aquilanti L., Taccari M., Garofalo C., Polverigiani S., Clementi F., Franciosi E., Tuohy K. (2018). Microbial dynamics of model Fabriano-like fermented sausages as affected by starter cultures, nitrates and nitrites. Int. J. Food Microbiol..

[69] Pasini F., Soglia F., Petracci M., Caboni M.F., Marziali S., Montanari C., Gardini F., Grazia L., Tabanelli G. (2018). Effect of fermentation with different lactic acid bacteria starter cultures on biogenic amine content and ripening patterns in dry fermented sausages. Nutrients.

[70] Coloretti F., Chiavari C., Poeta A., Succi M., Tremonte P., Grazia L. (2019). Hidden sugars in the mixture: Effects on microbiota and the sensory characteristics of horse meat sausage. LWT-Food Sci. Technol..

[71] Coksever E., Saricoban C. (2010). Effects of bitter orange albedo addition on the quality characteristics of naturally fermented Turkish style sausages (sucuks). J. Food Agric. Environ..

[72] Aleson-Carbonell L., Fernández-López J., Sayas-Barberá E., Sendra E., Pérez-Alvarez J.A. (2003). Utilization of lemon albedo in dry-cured sausages. J. Food Sci..

[73] Pini F., Aquilani C., Giovannetti L., Viti C., Pugliese C. (2020). Characterization of the microbial community composition in Italian Cinta Senese sausages dry-fermented with natural extracts as alternatives to sodium nitrite. Food Microbiol..

[74] Comi G., Muzzin A., Corazzin M., Iacumin L. (2020). Lactic acid bacteria: Variability due to different pork breeds, breeding systems and fermented sausage production technology. Foods.

[75] Dias I., Laranjo M., Potes M.E., Agulheiro-Santos A.C., Ricardo-Rodrigues S., Fialho A.R., Véstia J., Fraqueza M.J., Oliveira M., Elias M. (2020). Autochthonous starter cultures are able to reduce biogenic amines in a traditional portuguese smoked fermented sausage. Microorganisms.

[76] De Filippis F., La Storia A., Villani F., Ercolini D. (2013). Exploring the sources of bacterial spoilers in beefsteaks by culture-independent high-throughput sequencing. PLoS ONE.

[77] Stellato G., Utter D.R., Voorhis A., De Angelis M., Eren A.M., Ercolini D. (2017). A few *Pseudomonas* oligotypes dominate in the meat and dairy processing environment. Front. Microbiol..

[78] Bonomo M.G., Cafaro C., Salzano G., Zdolec N. (2016). Application of molecular methods in fermented meat microbiota: Biotechnological and food safety benefits. Fermented Meat Products: Health Aspects.

[79] Iacumin L., Manzano M., Panseri S., Chiesa L., Comi G. (2016). A new cause of spoilage in goose sausages. Food Microbiol..

[80] Stanborough T., Fegan N., Powell S.M., Tamplin M., Chandry P.S. (2017). Insight into the genome of *Brochothrix thermosphacta*, a problematic meat spoilage bacterium. Appl. Environ. Microbiol..

[81] Corral S., Salvador A., Flores M. (2013). Salt reduction in slow fermented sausages affects the generation of aroma active compounds. Meat Sci..

[82] Fonseca S., Gómez M., Domínguez R., Lorenzo J.M. (2015). Physicochemical and sensory properties of Celta dry-ripened “salchichón” as affected by fat content. Grasas y Aceites.

[83] Kumar P., Chatli M.K., Verma A.K., Mehta N., Malav O.P., Kumar D., Sharma N. (2017). Quality, functionality, and shelf life of fermented meat and meat products: A review. Crit. Rev. Food Sci. Nutr..

[84] Wood J.D., Enser M., Fisher A.V., Nute G.R., Sheard P.R., Richardson R.I., Hughes S.I., Whittington F.M. (2008). Fat deposition, fatty acid composition and meat quality: A review. Meat Sci..

[85] Weiss J., Gibis M., Schuh V., Salminen H. (2010). Advances in ingredient and processing systems for meat and meat products. Meat Sci..

[86] Mataragas M., Bellio A., Rovetto F., Astegiano S., Decastelli L., Cocolin L. (2015). Risk-based control of food-borne pathogens *Listeria monocytogenes* and *Salmonella enterica* in the Italian fermented sausages Cacciatore and Felino. Meat Sci..

[87] Gounadaki A., Skandamis P., Drosinos E.H., Nychas G.J.E. (2007). Effect of packaging and storage temperature on the survival of *Listeria monocytogenes* inoculated postprocessing on sliced salami. J. Food Prot..

